# Supplementary material for: Community Assessment for a Low-Carbohydrate Nutrition Education Program in South Africa
Source: Nutrients. 2022 Dec 23;15(1):67. doi: 10.3390/nu15010067 (PMC9824893; doi:10.3390/nu15010067)
Supplement: Supplementary file 1 [file nutrients-15-00067-s001.zip › Supplement_S1.pdf]

Supplement: Focus group discussion semi structured questions

- How would you describe your current diet?
- Can you describe the type of foods and drinks that you generally consume?
  - What is the must-have staple foods in your household?
  - What are the reasons why do you eat these foods?
- How would you best describe healthy living?
  - What things would healthy people do or how would they behave?
  - What do you and others do to stay healthy?
- What does healthy eating mean to you?
  - What kind of food do you consider as healthy?
  - What kind of food do you consider as unhealthy?
- Could you tell us about how the food you eat is generally prepared?
  - Who does the cooking in your household?
- Where do you usually go to buy most of your food and drinks?
  - What aspects influence your food choices (quality, budget, distance)?
- What do you understand as the Banting/low carb diet?
- What are your thoughts about Eat Better South Africa?
- How willing do you think women from your community will be to enrol in an education program to make dietary changes to improve their health?
- What are your thoughts on enrolling yourself in such an education program (to make dietary changes to improve your health)?
  - Why would you take part in the program?
  - What are the things that make it difficult to make dietary changes?
  - What are the things that may make it easy to make dietary changes?
