# Supplementary material for: Community Assessment for a Low-Carbohydrate Nutrition Education Program in South Africa
Source: Nutrients. 2022 Dec 23;15(1):67. doi: 10.3390/nu15010067 (PMC9824893; doi:10.3390/nu15010067)
Supplement: Supplementary file 1 [file nutrients-15-00067-s001.zip › Supplement_S2.pdf]

File S2.

Recommendations for EBSA to optimize the nutrition education program.

Recruitment	Previous experiences	Previous EBSA participants reported having improved their health, so the potential health benefits could be used to advertise and recruit participants [26].
	Clinics	EBSA should consider collaborating with the local clinics and engage with medical doctors and nurses [26].
	Community groups	Previously, some of the programs were offered to women who were already taking part in a physical activity group, which was free [26]. EBSA could look for those groups and other groups already created within the community. EBSA could look for collaborations with organizations that work already in the community (as the ones that were contacted to recruit participants for the current study).
Program's structure	Sessions' format	The EBSA educator in Ocean View, who was an actress, was seen as someone very approachable. This suggests that the sessions should be interactive and engaging for women [26].<br>The number of sessions created disagreements between the group of women, especially in Atlantis. Instead of providing one session every week, introduce a gap between sessions: for instance, provide two sessions per month [26].<br>As some women mentioned, poor weather conditions are likely to deter them from attending sessions [26]. The sessions should be in the mornings, since women prefer to spend the afternoons with family, andwhen it is not winter.
	Reduce emphasis on Weight loss	Some women felt that the program's main outcome was to lose weight. It is recommended to encourage women to be healthier while acknowledging participants' success. In that case, more aspects of the program should be considered for awards, such as best food recipes, best health markers, or best adherence to the diet [26].
	Privacy	Some women felt uncomfortable being measured in front of other participants and having their results visible to everyone. Although this was not the primary concern for most women,EBSA should conduct assessments in a more private manner [26].

	Language	The EBSA sessions should use a language accessible to most of the community. The EBSA educator should speak the languages that most community members can understand [26].
	Clothing	One of the challenges that some women faced during the program was that their clothes no longer fit them, and they could not afford to buy new clothes, because they were losing weight rapidly [26]. EBSA should take into consideration that some women might lose weight very quickly and that they need to find places to buy new affordable clothes.
	Meal plans	One of the concerns of food availability is the products' stock. Women explained that since they started the recommended diet simultaneously and were usually the ones that buy the groceries at home for the whole family, some of the products were out of stock in their local supermarkets [26]. It is recommended that EBSA creates different meal plans for participants each week.
	Physical activity	Women said they felt more energetic after following the EBSA diet, which leads to a desire to exercise [26]. As seen in the previous study]26] and this study these communities, where gang violence has increased in recent years, EBSA could consider providing women with some exercise programs that could be done at home in a safe environment.
	Mental health	Women mentioned during the community assessment that they felt they were out of control with their dietary habits and that stress sometimes made them eat compulsively, which could mean that because of different life struggles, they use food as a stress relief or a way out. EBSA could create a session related to mental health, how to deal with stressful situations, and hunger control to help women deal with these situations.
Follow-ups	EBSA coach	The coach's role is a necessary tool for EBSA's sustainability within the community as seen previously [26]. In new communities, EBSA should identify from the group of women, a woman that appears to have the best profile to become the next EBSA coach for the community. Considering that unemployment rates and retrenchment among women in those communities are high, it should be essential to know about women's situations and choose someone with whom they feel comfortable.
	Scheduled follow-up	Follow-ups provided by EBSA should be more consistent and planned, while in the program's development stage [26]. Some of the topics for the EBSA follow-ups can be how to maintain weight, understanding the health markers, how to access Banting resources (social media, EBSA/TNF

		website), how to 'easily' explain the recommended diet (for them to spread the word in the community).
Publicity	Doctors and nurses	Some women were advised not to follow the Banting diet by their doctors [26]. EBSA could invite doctors and nurses to one of the EBSA sessions for them to see what the program is about. EBSA should also investigate local health care professionals' perceptions of LCHF, and EBSA.
	Family and friends	Many women reported that they had little or no support from family, friends, or colleagues at the beginning of the program, although some experienced increased support when these people could see results [26]. Some women also tried to encourage other people to join the program or follow the diet but struggled to convince them [26]. Moreover, women from the community assessment acknowledged that one of their greatest challenges was their families' support. By having a session where participants can invite people that are usually around them, the program and diet can be explained to these people, and by doing this, women might receive more support from their networks.
	Media	EBSA could promote the program and the recommended diet on the local radio and in newspapers (maybe with before and after pictures of previous participants) [26]. It could also be an excellent way to follow-up with the community. EBSA could provide meal plans once a month to the newspaper and other resources to keep women informed.
